# Traveling Letters from the Senior Editor

**Published:** 1847-09

**Authors:** 


### THE WESTERN JOURNAL.

New Series.—Vol. VIII.—No. III.

LOUISVILLE, SEPTEMBER 1, 1847.

### TRAVELING LETTERS FROM THE SENIOR EDITOR.

NUMBER ONE.

*Mountains of Western Virginia,*
July 9th, 1847.

*To My Colleagues:*

Gentlemen:—If you should mark this letter No. 1, you might involve me in some difficulty, as I may not find time or topics for another. Even this, I fear, will possess but little interest, and I charge you not to publish it, to the exclusion of the communication of *any* correspondent. We must regard the interests of our readers, and not give them bad matter, if good be within our reach. After this commendably modest exordium, I proceed to show that the charge I have just given you is very timely.

Leaving the Pittsburgh packet, *Hibernia*, (which I can strongly recommend to all our traveling brethren, as the famine which pervades her namesake-country finds no place at her table), I took the stage at Guyandotte, and set off for Charleston and the salines of the Kenawha Valley. On the way I stopped, for a moment, at Coal Bridge, to see

### *A Lady said to have been Cured of Fungus Hæmatodes.*

The case is not unworthy of a passing notice. The patient, in the latter part of last summer, or early in autumn, was brought to Cincinnati by her husband, for the purpose of consulting Dr. Rives, who, after an examination, desired me to see her. We found her left breast enormously enlarged and tuberous; some parts were much softer than others; portions of it had a dark red color; and the veins of the skin in many parts were varicose. The tumor had been several years in arriving at this condition. It was the seat of occasional pain, and the general health of the patient was impaired. In short, we concurred in the opinion that it was a *fungus hæmatodes*, and that excision was inadvisable. Under this diagnosis and prognosis, we recommended to her to return and do nothing. She left us, but instead of going home, visited the neighboring town of Gallipolis, in Ohio, and put herself under the care of a practitioner who, it was said, could cure all sorts of cancers, without an operation. In the ensuing winter I received a newspaper, containing a letter from the lady, who is a respectable teacher of girls at Coal Bridge, stating that she was well. The publication, I confess, took me by surprise, while it afforded me great pleasure. It was to ascertain by personal observation her exact condition, that I stopped at the place just mentioned.

I found that her breast was entirely gone, and the skin over the part apparently sound and healthy. The eschar was somewhat extensive and spragling, and felt rather hard; but she said it was entirely free from pain and all morbid sensibility. There were no axillary tumors, but I am not quite certain that there were any last summer. Her appearance was healthful, and she assured me that she had never felt better in her life. The account she gave me of the treatment was, that the doctor, whose name I have forgotten, began by making a hole in the breast with caustic, into which he put something which killed, successively, the whole tumor. The dead parts he dissected out with scissors, from time to time and in the aggregate they weighed eleven pounds! During this treatment, which lasted for several weeks, she experienced considerable pain, which was alleviated with sulphate of morphine. After all the diseased structure was removed, granulation and cicatrization proceeded rapidly; and when I saw her, at least six months had elapsed without any symptom of recurrence of the disease, in that or any other part.

Now, if Dr. Rives and I were mistaken in the diagnosis of this tumor, and it was neither carcinomatous nor encephaloidal, it was at least a very large and painful chronic induration of the breast; and although the removal of such a non-malignant tumor with the knife would have been a preferable mode, still we see that it may be accomplished successfully with caustic. But I cannot give up the opinion, that it was malignant; and under that conviction am led to indulge in a few speculative remarks.

In all cases where cancer or encephaloid is the offspring of a constitutional diathesis, the removal of the tumor, either by incision or any kind of caustic, must of necessity be unavailing; but if there be cases not preceded by such a diathesis, extirpation may be permanently successful. The canons of surgery direct that for this purpose the knife should be preferred to caustic; but is this certainly correct? The object of the operator is to remove the whole of the diseased *structure*, but a diseased *action* precedes and causes the morbid structure, and that kind of action in a germinal or incipient stage may exist in parts which show no pathological change, and thus render the operation unavailing. Now, may it not be—may it not happen—that among the various kinds of caustics and escharotics, there is one which can destroy both the pathological structure and the pathological action, and thus achieve what the knife cannot? I confess that I am not without hope, that this question will sooner or later be answered in the affirmative. But experiment is necessary to an answer of any kind; no regular member of the profession, however, is much inclined to such experiments because they tend (improperly) to place him, in the estimation of his brethren, in the category of mere empirics. Such being the public opinion of the profession, why should we discourage our patients from placing themselves in the hands of the latter? The only objection to their doing so is, that tumors and ulcers which are non-malignant, may be aggravated by the harsh treatment; but in most, perhaps all cases, regular ph, sicians are consulted before the cancer-doctors, and may then declare the diagnosis. Moreover, it is a painful and unsatisfactory thing to a patient to be told that his disease is irremediable, and that he will live longer to do nothing than to do anything. Few hearts are stout enough to endure and patiently wait on such a course. It is better, therefore, for the happiness of the patient to be doing something; and if what is done should sometimes shorten life, he loses very little. Referring then to the *possibility* of a valuable discovery, and to the cheerfulness of the unfortunate victims of cancer and encephaloid, I can see no objection to their consulting empirics. At the same time, I am bound to say, that the majority of that class are mere impostors, and use nothing but what the regular profession have long since tested and found ineffectual. It is not my intention to class with such the individual who treated the case of which I have spoken, for I neither know him, nor the agent which he employed.

### *Kenawha Salines.*

An evening ride of two hours up the beautiful Kenawha Valley, carried me from Coal Bridge to Charleston, the commercial emporium of the salt-works. Above that town the artesian salt-wells and furnaces for evaporation, extend up both sides of the river for ten or twelve miles.

The borings are of various depths, from 300 to 1200 feet. The water ascends in them, not so much by a source in higher ground, as the force of escaping carburetted hydrogen gas; and thus we have here on a great scale, the same phenomena which occur, in miniature, on the small island at the Balize, where carburetted hydrogen, developed in the alluvium carried down by the Mississippi, forces up feeble veins of water, much salter than that of the surrounding gulf at its surface, which is diluted with river water. The evolution of gas in the different Kenawha wells is very unequal. In some the quantity is so great, and it issues with such force, as to throw a column of water many feet in height into the air, when there is no external obstruction. At one or more of these borings the gas is collected in gasometers, and then suffered to escape under the boilers where its ignition produces an intense heat, altogether sufficient for the evaporation of the water which is forced up; thus presenting one of the most beautiful arrangements which the science of economics (if there be such a branch) could afford. But the furnaces generally are heated with bituminous coal, of which the adjoining hills afford an abundance. These hills are from five hundred to seven hundred feet above the level of the river, and deeply cut with narrow ravines, many of which open laterally into the principal valley. About sixty or seventy gallons of the brine afford a bushel of salt, and from the quantity manufactured, near one hundred and eighty millions of gallons of water must be annually converted into steam. This steam no doubt carries up, in solution, a portion of salt and other soluble matters. Here then we have a peculiar locality: 1. The constant escape of carburetted hydrogen. 2. The disengagement of a great amount of caloric. 3. The production of immense volumes of stone-coal smoke. 4. The ascent of vast quantities of a gaseous vapor holding saline matters in solution. 5. A heated atmosphere near the kettles, to which the operatives are at times greatly exposed. These operatives, chiefly negroes, are so numerous as to be counted by the thousand. Such are the conditions to which they are exposed, and to ascertain the effects of those conditions on their constitutions and health, was the chief object of my visit. On this point I shall not go into details, but merely remark, that the employment is regarded as decidedly salubrious.

A word on the meteorological phenomena of this spot. 1st. In calm summer evenings the air cools more rapidly on the adjoining hill tops than in the valleys, and rolling down to the intervening ravines, makes its way, in refreshing breezes, into the principal valley; a phenomenon which I have observed in other parts of this rugged country. 2d. According to the Espyan theory of rain, should we not expect frequent showers in summer over this spot? One person with whom I conversed, declared them to be more frequent than in the surrounding country, but attention had not been so generally turned to the subject as to supply facts for a decision.

### *Pomeroy Coal Mines.*

The voyage down the beautiful Kenawha, when the water is low, is over a series of pools and ripples. Its banks are so high that inundations of most of them are unknown, and autumnal fever is correspondingly rare, until we come near its mouth; where the alluvial grounds are somewhat more pondy. The Pomeroy coal banks, in the State of Ohio, are fifteen miles above the mouth of the Kenawha. The excavations are all horizontal and on the same plane, which is far above high water mark of the Ohio. The principal drift has been carried through a hill, so as to establish a current of air from south to north. Continuing far into but not through, another ventilation is effected by chimneys carried up to the surface, in which fires are kept burning. Thus the air of the mines is carried out, and the accumulation of mephitic gases prevented. In one part, however, our candles burned dimly, except when elevated to the upper part of the drift, indicating the presence of carbonic acid. No 'firedamp' explosion has happened. Gun powder is employed in blasting the coal, and thus the air is rendered more or less impure by the gases which it generates. All the excavations were dry. The temperature of one of the most secluded was 58°.5, but in general they were 60° or a little more. Nearly one hundred miners are employed in these banks, where they commonly pass about twelve hours of the twenty-four working by the light of candles and lamps, often in very constrained positions, and breathing a great deal of coal-dust. All the operatives with whom I conversed, as well as the enterprizing owners of the establishment, declare that they are a healthy race of men. This they ascribe in part to their working in an atmosphere so uniform in temperature.

### *Marietta.*

From Pomeroy I proceeded (again in the Hibernia) to the *ancient* town of Marietta, at the mouth of the Muskingum river. Here began the settlement of the State of Ohio on the 7th of April, 1788. Till within four years of that time, the present States of Kentucky and Ohio were portions of the 'Old Dominion.' With great propriety, therefore, may they be called sister States, and their physicians medical *brethren.* I have spoken of Marietta as an *ancient* town, and so it is, if we reckon by the number of inhabitants—two millions—which now swarm in the State of which it was the beginning; but if we look to its years, we shall find that it cannot boast of as many as two of the Faculty of our University. Its size, however, is not very great, and while Cincinnati, of later development in the political organism, is tending to hypertrophy, Marietta has long shown signs of atrophy. Nevertheless, her structure is, and has been, normal and healthy; and she has long had two physicians who have conferred honor on her name. They are Dr. HILDRETH and the late Dr. COTTON.

Dr. Hildreth left his native State, Massachusetts, and settled in this place forty years ago. His talents and acquirements were respectable, but the strength of his character, and the earnest it gave of future distinction, lay in its *morale*. He formed, and has perseveringly carried out, the determination to employ that great capital, TIME, to the best advantage. Hence he has constantly kept important objects in view, and thus achieved more than ninety-nine-hundredths of his western cotemporaries, either north or south of the Ohio. All, it is true, has not been professional, but of that which is strictly so, he has done much more than most of us who have aimed at nothing else. He has published many important papers on the diseases of his locality, and has many folio volumes in manuscript of recorded experience, out of which to draw materials for future publications. For a quarter of a century he has kept and published, annually, in Silliman's Journal, a meteoroiogical register, with interesting notices of epidemic diseases, and facts for a *Calendarium Floræ*, of the different seasons; he has described the great coal basin in which I am now traveling; he has made a record of the curious antiquities of the spot on which he lives; lastly, he has written the early history and biography of the region round about him, both in Ohio and Virginia. Such are the fruits of good purpose, temperance, piety, and unfaltering industry; and the old tree is still succulent and vigorous enough to bring forth many more. Now, could I perform a kinder office to our young readers than to present them with the example of such a man? I trust they will believe I could not. May they henceforth resolve to do likewise.

Dr. John Cotton, the cotemporary and friend of Dr. Hildreth, is now no more, except in the hearts of those who knew him, or had, through his successful practice of 30 years, been rescued from the hand of the destroyer. His emigration from Massachusetts was in the year 1816—his death in the month of April last. My acquaintance with him was limited, but I knew his character well, and have before me the data for a just estimate of it, in a publication made immediately after his death by his *confrere*, Dr. Hildreth. One of his intellectual characteristics was the care and success with which he cultivated the Latin classics; so that up to the time of his death he continued to compose in that language, with a degree of accuracy and elegance, which secured the admiration of all around him, in a community where there are many good scholars. He was also acquainted with the Greek language, and at the age of forty studied the Hebrew. His attainments in astronomy were respectable, and rendered useful to the community by his delivering courses of public lectures on that science. His taste took, likewise, the direction of political and legal science. For one session he served in the Legislature of Ohio, and for twenty-one years was an associate judge of the county in which he lived. One of the founders of the Marietta Collegiate Institute, he was from its beginning a trustee, and for some years the president of the board. Sincerely and devotedly pious, he was indefatigable in every religious duty, and never failed (except when prevented by professional business) to be at his post as a Sunday-school teacher. But none of these things prevented him from rearing a professional reputation, which for years gave him a distinguished place among the leading practitioners of the section of country in which he lived; and had his life been prolonged, he would, no doubt, have made a liberal contribution to our western medical literature, for his application was great, his love of truth sincere, his judgment admirably balanced, and his sagacity acute. Well would it be for society and the dignity of our profession, if every town of two thousand inhabitants contained such a man.

I must skip from the mouth of the Muskingum, in Ohio, to the escarpments of the Laurel mountains in Virginia, where some things of interest present themselves, but as my letter is already too long unless it were better, I shall give you only one.

### *A Splendid Practice to be Sold out Cheap.*

In the neighborhood of 'Cheat River,' my host, whose dimensions and complexion marked him as a lineal descendent of good old Mr. Boniface, happened to be a doctor. While taking a 'hasty' cup of tea, with stewed cherries and bread and butter (a dinner which I recommend to all travelers) my inquiries disclosed to him my own profession, and he soon became pleasantly communicative. After assuring me that ague and fever is unknown in that region, he proceeded to state that he wished, henceforth, to devote himself exclusively to innkeeping; and that, having a '*splendid practice*,' he was desirous of selling out to a successor. The temptation was very strong; but as I wished not to be *cheated* out of my visit to Quebec, I made an early escape from the scene of allurement. As my two-horse stage, with no passenger but myself, trundled quietly along over mountains made still loftier by a panoply of oaks and chestnuts, intermingled with pines; or through deep and wild valleys, 'darkly smiling' with Kalmias and Rhododendrons, and fanned by delicious breezes; I could not but feel sorry for many of our toiling and wilted brethren in the sunny and sultry south, and resolved to apprize them of this attractive spot. To the young man who has sauntered among the tributaries of

Pearl river, without finding pearls; or, instead of black patients, on the wide bottom of the Black Warrior, has contracted the 'black janders;' or, to the still more luckless wight, on the banks of the Tallapoosa, who, aiming to get a true *rib* with a large cotton-field, has got within his false rib a large spleen; this retreat from all sorts of miasms, agues, and congestives, with the immediate prospect of a 'splendid practice,' offers advantages too good, I am sure, to be neglected. I would like to expatiate upon them, but having already extended my letter to an unreasonable length, I must request you to do it in an editorial.

Your friend and colleague,

DAN. DRAKE.

---

### TRAVELING LETTERS FROM THE SENIOR EDITOR.

NUMBER TWO.

*Mountains of Pennsylvania,*

July 28th, 1847.

*To my Colleagues:*

Gentlemen:—Although nearly three weeks have elapsed since the date of my first letter, I fear that I have not picked up sufficient *materiel* for a second; nevertheless I will begin. Should it turn out to be shorter than the last, our readers will not be displeased, however much our compositors may grieve, seeing that I write a plain, large, honest, readable hand, so unlike the illegible chirography of many of our correspondents over which the poor printers are obliged to boggle and blunder. How strange it is that penmanship has never been made a branch of *medical* education. I wonder the late National Convention did not give attention to the matter. Half of our physicians write so carelessly, or awkwardly, or affectedly, that the whole Champollion school could not decipher their hieroglyphics. To say nothing of communications for the Journal which cannot be published because they cannot be read either forward or backward, and are therefore lost to the world, I may refer to prescriptions, which, on the files of the compounding apothecaries of Cincinnati and Louisville, present specimens of the obscure which are truly original. How many poor patients have had their diseases aggravated, or their lives placed in jeopardy, by this heterologous penmanship, no one can tell. Even death has been its effect.

I knew a physician to order, in a recipe put up by one of his own pupils, a certain quantity of water, and intending to write *Aq. font.*, he wrote what was deciphered as *Aq. fort.*—the child took a dose of the mixture, and immediately expired. So much for making an *n* look like an *r*. In their directions to patients, moreover, there is often great looseness, even in punctuation, an instance of which I may mention, to dissipate the feeling of sadness which the last is so well fitted to raise.

A physician wrote to his patient—'*Take wine, and bark,*' whereupon the poor fellow swallowed a glass of the former, and fell to barking! One other example will conclude this unpremeditated exordium. A physician made out his bill, and wrote at the bottom, '*Please pay,*' to which he subscribed his name. As his patient, like many others before and since, had more acuteness than cash, he discovered that the word *Please* was so written that it might be read *Received,* and gave no attention to the matter. In due time he was brought before a Justice of the Peace, when he produced the account. His worship, after a deliberate examination, in which he was assisted by his wife and the constable, decided that it was a receipt, and the poor doctor had to pay the penalty of his carelessness, by paying the cost, besides losing seven dollars and fifty cents, the charge for his services!

But I must turn from things common to the whole Valley of the Mississippi, to those which are found in a small section of its illimitable borders.

Soon after my last date in Virginia, I entered Pennsylvania through the southern county, Fayette, over the Laurel mountain; and am now in the northern border connty of Warren, near New York, having traversed the State from south to north, in its sub-alpine belt. South of Pittsburgh, my rambles were in the basin of the Monongahela; north, in that of the Allegheny, on the banks of which, two hundred miles above the city, I am now writing. On this trip I have met with many objects, scenes, and events, which would interest our readers as *men*, but as a medical journal is not the vehicle in which to send them forth, they will, I fear, be lost to the world. Happily the world will never know its loss, and therefore will not grieve.

While at Uniontown, in the Valley of the Monongahela, I made a visit with Mr. Oliphant to his noted Iron-works—bearing the liberal name of '*Fair-chance.*' They are at the foot of Laurel mountain, within the coal measures, and present a rare assemblage of natural facilities. Ore (of which I brought away specimens for the cabinet of our University), coal for the manufacture of coke to be used in smelting, limestone the only required flux, and fire-clay for the lining and hearths of furnaces, all lie *stratum super stratum*, in low rounded hills; while water, issuing from the side of the mountain, descends and is distributed by pipes throughout the works. Both the ore and limestone are in oval, circular, or reniform, compressed nodules; and much of the latter, is to some extent impregnated with the former.

In roasting and smelting the ore the operatives throw it into the top of a chimney built on the hill side, and are much exposed to whatever fumes may be driven off by the heat below. In questioning them concerning the effects of these fumes on their health, they answered that sometimes their breathing was affecteed and they felt faint. One of them added, that he had lost a great deal of flesh, and was subject to sudden and transient colicky pains. A good deal of sulphur, combined or uncombined, is no doubt exhaled, and perhaps some arsenic, but on this point I have no facts. The men who melt the pig metal and make bar iron, are not exposed to these exhalations, but they work in a temperature which excites profuse perspiration. I found it to vary in different parts of the establishment from 90° to 105° of Fah. They appeared to enjoy good health.

The country between Uniontown and Pittsburg, by way of Brownsville and Washington, is, perhaps, the oldest settled portion of the west, except some of the interior counties of Kentucky. It is subject to remittent autumnal fever, in a sporadic degree, but intermittents are nearly unknown. Lately a well characterized typhoid fever has prevailed, as a local epidemic, in several localities; and dysentery appears to be more frequent than farther west. The surface is every where dry and hilly.

In one of the towns I met with a very intelligent physician, who, mischievously, amused himself by telling me of an apparatus for fractures of the thigh, which had been patented and hawked through that region, in connexion with the certificates of several medical *professors.* On examination, he pronounced against it, *a priori*; but one of his neighbors rejected his opinion, and applied it to the broken thigh of a man—which, when ossific union was effected, proved to be two inches shorter than the other! From this it appears, *mirabile dictu,* that medical professors are not infallible.

Speaking of patent apparatus reminds me of patent medicines and quackeries in general, on which I would remark, that the Western Pennsylvanians do not appear to be as gullible in reference to them as the people in some other quarters. A druggist in Pittsburgh, it is true, has made a large fortune by some kind of worm killer, but systematized steameopathy, homœopathy, and hydropathy have not flourished there as in Cincinnati and Louisville. The homœopathist, who, a few years since, almost reduced the physicians of Louisville to homœopathic doses of bread and meat, had previously 'tried his hand' on the good people of the *Iron* city, who proved to be too *hard* for him. In fact they *tired* him with homœopathic doses of the staff of life, when he *rolled* off for the Falls' City. As you *know,* like another dog he there 'had his day,' and then disappeared; but perhaps you do *not* know where he next made a stand. It was on the banks of the Tombigbee, in the State of Mississippi, where he *rolled* over the steameopathists, who had previously scalded out the 'regular doctors!' But as our readers would no doubt prefer homœopathic doses of this kind of gossip, I will proceed to something else.

The Iron City cannot be said, in any sense of the word, to make an

*imposing* appearance, for its exterior is dingy and sooty, and its intrinsic merits are so great that it is found more excellent than it appears. I hope, in due time, to receive an accurate estimate of the amount of bituminous coal consumed daily within its extended limits, till when, I shall only say, that the quantity is greater than in any other city in the Union, while its topography is by no means favorable to free and full ventilation; as a hill, nearly five hundred feet high, stretches along its southern side, and the Ohio, on leaving it, takes a course very much to the north-west. Thus, its atmosphere is forever charged with the mechanical and gaseous impurities which arise from its 'thousand and one' artificial volcanoes. The inhabitants believe all this highly beneficial in the prophylaxis of epidemic diseases, and refer to the absence of ague and fever, and their slight invasion of epidemic cholera, for proof. I shall admit the facts, without at present either adopting or rejecting their theory; but must say, that if they escape many of the violent diseases of other localities, they do not present the aspect of high health and abounding vigor. The corpulent rotundity so common in the streets of the Falls' City, is not seen here; and a sallow hue, with somewhat of contracted features, is common. Even the children are not chubby and ruddy, but present many lean faces, and older visages than properly belong to their years. Of actual diseases generated by the perpetual inspiration of the factitious atmosphere, I hope to say something 'at another time and place.'

You are aware that goitre was once epidemic in Pittsburgh, and has disappeared. Before going thither, I had assumed that certainly *I* could find out the cause, but, alas! how often Mr. *I* disappoints our expectations. It is *consoling*, however, to know that the very respectable medical gentlemen of that city have equally failed. Most of them, in fact, know nothing of the disease, except as a matter of history, for it began to cease more than thirty years ago. Dr. Denny is its only historian, and he wrote chiefly from tradition and the recollections of the 'oldest inhabitant.' But for his valuable paper, in the 9th vol. of the Philadelphia Journal, its history would be most imperfect. At present it prevails less in the city than in the country and the country towns, some of which, as Franklin and Warren on the Allegheny river, present a considerable number of cases, more than any part of the west which I have visited, except perhaps a zone across the State of Ohio, embracing the Sciota and Cuyahoga vallies.

I am now writing in the midst of the pine-lumber region of the Valley of the Mississippi. There is no other which can compare with it in extent or the number of operatives employed. It comprehends six or eight counties in Pennsylvania, with three or four in New York; and abounds in mountain streams, on which there are saw-mills innumerable, with quite as many trout and water-falls. Of the diseases which prevail among the laborers in such a locality, one cannot find much to say. They are chiefly the phlegmasiæ of the lungs and joints. Here, then, is the place to which dyspeptics and hypochondriacs from the low country should repair, to fish and hunt among whortleberries and rattlesnakes, and clamber over the rugged hills of subcarboniferous conglomerate, or cut *down* and saw *up* the gigantic white pine, or drag it to the mill with oxen, or raft the boards—according to their respective tastes or pecuniary means.

To-morrow I shall enter the Empire State and still be within the waters of our own beautiful Ohio, whose cephalic dimensions are such, that if he could shake his head, the mountain rivulets would be agitated from New York to Alabama, through eight degrees of latitude.

Your obedient servant,

